# Early-Onset Diabetes as Risk Factor for Pancreatic Cancer: miRNA Expression Profiling in Plasma Uncovers a Role for miR-20b-5p, miR-29a, and miR-18a-5p in Diabetes of Recent Diagnosis

**DOI:** 10.3389/fonc.2020.01567

**Published:** 2020-09-11

**Authors:** Francesca Tavano, Andrea Fontana, Tommaso Mazza, Domenica Gioffreda, Tommaso Biagini, Orazio Palumbo, Massimo Carella, Angelo Andriulli

**Affiliations:** ^1^Division of Gastroenterology and Research Laboratory, Fondazione IRCCS Casa Sollievo della Sofferenza, Foggia, Italy; ^2^Unit of Biostatistics, Fondazione IRCCS Casa Sollievo della Sofferenza, Foggia, Italy; ^3^Unit of Bioinformatics, Fondazione IRCCS Casa Sollievo della Sofferenza, Foggia, Italy; ^4^Division of Medical Genetics, Fondazione IRCCS Casa Sollievo della Sofferenza, Foggia, Italy

**Keywords:** pancreatic cancer, circulating microRNA, early diabetes, expression profile, diagnostic performance

## Abstract

The high prevalence of early-diabetes in patients with pancreatic cancer (PanC) implies that its recognition could help identify people at high risk of developing PanC. Candidate microRNAs (miRNAs) associated with recent diabetes were screened from our previous miRNA expression profiling on 10 pools of plasma from PanC patients and non-PanC controls, both including also subjects with early- and late-diabetes. The droplet digital PCR (ddPCR) was used to re-test candidate miRNAs in a new independent cohort of 69 subjects (40 PanC, 29 non-PanC) with early- (17 PanC, 13 non-PanC) or late-diabetes (23 PanC, 16 non-PanC), and in 100 non-diabetic healthy subjects (HS). miRNA levels were evaluated for differences between subjects enrolled into the study and for their diagnostic performance, also compared to the CA 19-9 determinations. MiR-20b-5p, miR-29a, and miR-18a-5p were selected from the previous miRNA expression profiling. The ddPCR confirmed the increase of miR-20b-5p and miR-29a levels in PanC with early- compared to those with late-diabetes. Conversely, miR-20b-5p, miR-29a, and miR-18a-5p were over-expressed in both PanC and non-PanC with recent diabetes compared to HS, and each miRNA achieved a similar diagnostic performance in distinguishing either PanC or non-PanC with early-diabetes from HS (miR-20b-5p: AUC = 0.877 vs. AUC = 0.873; miR-29a: AUC = 0.838 vs. AUC = 0.810; miR-18a-5p: AUC = 0.824 vs. AUC = 0.875). Despite miR-20b-5p and miR-29a expressions were also higher both in PanC and non-PanC with late-diabetes with respect to HS, the diagnostic accuracy in PanC with late-diabetes vs. HS reached by each miRNA (miR-20b-5p: AUC = 0.760; miR-29a: AUC = 0.630) was lower than the ones achieved in PanC with early-diabetes vs. HS. Furthermore, miR-20b-5p achieved a higher diagnostic accuracy to discriminate non-PanC with early-diabetes from HS (AUC = 0.868; SP = 81%; PPV = 32.1%) compared to the CA 19-9 (AUC = 0.700; SP = 40.0%; PPV = 15.5%), and the joint (miR-20b-5p and CA 19-9) discrimination ability was higher than the one achieved by the CA 19-9 tested alone (AUC = 0.900, *p* = 0.003). Our data highlighted the association between miR-18a-5p and early-diabetes, and suggested for miR-20b-5p and miR-29 a role in identifying early diabetes in PanC, albeit not as an early manifestation of cancer. MiR-20b-5p as more informative marker than CA 19-9 in distinguishing non-PanC with recent diabetes from HS was also uncovered.

## Introduction

The increasing diagnostic and therapeutic burden of pancreatic cancer (PanC) implies that early diagnosis represents the only opportunity to improve long-term disease survival. The absence of sensitive and specific markers for early detection of PanC, and the lack of a high-risk population for the development of the disease limit the possibility to screen people (1).

The very high prevalence of early-onset diabetes in patients with PanC and the experimental evidence suggest that diabetes is caused by cancer, thus recent diabetes could help define the group of subjects at high risk for developing the PanC. Diabetes occurs in almost half of all patients with PanC. The reported prevalence varies from 23 to 75% (2), and most cases of PanC-associated diabetes are of recent onset, beginning up to 2 years preceding the diagnosis of cancer (3–5). Humoral process mediates the pathogenesis of diabetes in PanC (6): β-cells are made susceptible to failure by circulating factors and the increase in insulin resistance associated with PanC is sufficient to cause β-cell decompensation and diabetes; in addition, the development of diabetes in PanC is probably a result of an interaction between clinical risk factors for development of diabetes and tumor factors, thus individuals with a predisposition to diabetes might develop diabetes more frequently in the setting of PanC than those without risk factors for diabetes. Overall, new-onset diabetes has been recognized as a manifestation of PanC and might play a role as a marker of early asymptomatic cancer (6–8). In addition, although with controversial results, the impact of diabetes on the long-term outcomes of patients with PanC has also been investigated (9–11). However, markers for early-onset diabetes associated with PanC have not yet fully investigated, and a better understanding of the pathogenesis of PanC-associated diabetes might uncover new promising clouds for early diagnosis of PanC.

In the attempt to test the relationship between diabetes and miRNA expression deregulation, different authors have investigated miRNAs levels alterations in the blood of diabetic patients compared to healthy subjects. Both newly diagnosed and long-term diabetic patients have been taken into account, and several miRNAs with increased (miR-320a, miR-142-3p, miR-222, miR-29a, miR-27a, miR-375) and decreased (miR-197, miR-20b, miR-17, miR-652) expression levels have been identified (12). More recently, the over-expression of a panel of six miRNAs (i.e., miR-483-5p, miR-19a, miR-29a, miR-20a, miR-24, miR-25) in serum has been reported to improve the diagnostic performance in distinguishing patients with PanC-associated diabetes from healthy controls (13).

This study aims to uncover novel miRNAs in plasma useful as biomarkers for recent-onset diabetes, potentially associated with PanC. To pursue this intent, we re-evaluated our previous data on miRNAs expression profiles in pools of plasma from PanC patients and no-PanC controls, both including also subjects with early- and late-onset diabetes (14). Candidate miRNAs discovered through the array-based approach were re-tested in an independent cohort of 69 subjects (PanC and no-PanC) with early- and late-onset diabetes, and in a control group including 100 healthy subjects without diabetes (HS). Differences in miRNA expression levels in subjects with early-diabetes compared to either those with late-diabetes or non-diabetic HS were evaluated. MiRNAs plasma levels were also tested for their diagnostic performance, and compared to the CA 19-9 determinations.

## Materials and Methods

### Study Design and Population

This study was designed following the same methodological approach described in our previous article (14). Briefly, two distinct experimental phases were conceived: the discovery phase, where miRNAs with a potential role in recent diabetes were screened by profiling miRNA expression levels in pool sets of plasma; the validation phase, where miRNAs emerged from the screening phase were re-tested in a new independent study population.

In the discovery phase, we started from our previous microarray data on 10 pools of plasma from PanC patients and non-PanC controls (ArrayExpress, Accession Number: E-MTAB-8378), including two pools from subjects with early-onset diabetes (Pool_4_ from non-PanC, and Pool_9_ from PanC) and two other pools from subjects with late-onset diabetes (Pool_5_ from non-PanC, and Pool_10_ from PanC) (14). Herein, a total of 169 subjects were enrolled into the validation phase, including 69 subjects with diabetes (29 non-PanC and 40 PanC) and 100 non-diabetic HS used as a control group. The median age of subjects within the diabetics and the controls was similar (67 years vs. 63 years, respectively). The former group included subjects with early diabetes (*N* = 30), i.e., with diabetes diagnosed within 2 years prior the samples collection (for non-PanC, *n* = 13) or the clinical diagnosis of PanC (for PanC, *n* = 17), and 39 subjects with a long-time diagnosed diabetes (16 non-PanC and 23 PanC). The control group included subjects who received a diagnosis of functional gastrointestinal disease (e.g., gastritis, gastro-esophageal reflux disease, dyspepsia, and somatic stress) at the outpatient clinic of the Division of Gastroenterology; as expected, subjects with chronic liver disease with or without cirrhosis, chronic pancreatitis or pancreatic cysts, inflammatory bowel diseases, tumors of the digestive system and biliary tract, polyps were not enrolled into the control population. In all the subjects, the presence/absence of diabetes was ascertained by medical history interview. Demographic features and baseline clinical-pathological characteristics of the validation cohort are shown in Table 1.

**Table 1:** Demographics characteristics of pancreatic cancer (PanC) patients, non-PanC controls and non-diabetic healthy subjects (HS), and baseline clinical-pathological features of PanC patients included in the validation cohort.

	**PanC**		**Non-PanC**		**Not diabetic-HS**
	**(*****N*** **=** **40)**		**(*****N*** **=** **29)**		**(*N* = 100)**
**Age** (years). median (IQR)	68.0 (63.5–76.0)		64.0 (65.0–72.0)		63.0 (57.0–70.0)
**Gender**. *N* Male (%)	20 (50.0)		9 (31.0)		43 (43.0)
**Pre-operative classification**. *N* (%)					
Resectable	8 (20.0)				
Locally advanced	18 (45.0)				
Metastatic	14 (35.0)				
**Surgery**. *N* (%)					
No	32 (80.0)				
Yes	8 (20.0)				
**Tumor location**. *N* (%)					
Head	28 (70.0)				
Body/Tail	12 (30.0)				
**Tumor stage**. *N* (%)					
IIA	2 (5.0)				
IIB	6 (15.0)				
III	18 (45.0)				
IV	14 (35.0)				
**Adjuvant therapy**. *N* (%)					
No	21 (52.5)				
Yes	19 (47.5)				
**Diabetes**, ***N***	**Early**	**Late**	**Early**	**Late**	
	**(*****N*** **=** **17)**	**(*****N*** **=** **23)**	**(*****N*** **=** **13)**	**(*****N*** **=** **16)**	
**Age** (years). median (IQR)	68.0 (35.0–70.0)	68.0 (63.0–76.0)	63.0 (61.0–64.0)	67.0 (61.0–74.0)	
**Gender**. *N* Male (%)	7 (41.2)	13 (56.5)	6 (46.2)	3 (18.8)	

All the subjects enrolled into the study were collected at the Divisions of Gastroenterology of “Casa Sollievo della Sofferenza” Hospital, San Giovanni Rotondo (Italy), after signing an informed consent form. The study was approved by the local Ethics Committee (Prot. No. 96/CE/2011).

### Plasma Collection, miRNAs Purification and Quantification by Droplet Digital PCR

Plasma samples were collected and processed for RNA isolation as previously reported (15). Absolute levels of miRNAs in plasma were quantified at the droplet digital PCR (ddPCR) system (Bio-Rad Laboratories, Hercules, CA) by using the TaqMan miRNA Reverse Transcription Kit and miRNA-specific stem-loop primers (Thermo Fisher Scientific, Foster City, CA; cat. No. 4427975; hsa-miR-18a-5p, Assay ID: 002422; hsa-miR-29a, Assay ID: 002112; hsa-miR-20b-5p, Assay ID: 001014) according to the manufacturer's instructions, and following the same experimental protocol described in our previous work (15).

### Data Analysis

Raw microarray data and miRNAs expression data were analyzed as previously reported (14). Statistical differences in miRNA expression between plasma pool sets were assessed using the ANOVA test, and bysetting a significance threshold to 0.01. Log-transformed and quantile normalized probeset signals of differentially expressed miRNAs were represented by heatmap, using *Pearson* correlation as a dissimilarity measure.

The Shapiro–Wilk and Kolmogorov–Smirnov tests were performed to assess whether the values of miRNAs expression levels and CA 19-9 serum marker were normally distributed. Because of the violation of normality assumption (having also tested the distribution of log-transformed values), expression levels of miRNAs and CA 19-9 serum marker were reported as median along with interquartile range (IQR, i.e., first-third quartiles) and pairwise comparisons of miRNA levels and CA 19-9 determinations between subjects with early diabetes and either those with late diabetes or non-diabetic HS were performed using the Mann–Whitney *U-*test. For each miRNA, and for CA 19-9 a boxplot was produced, along with raw data points (a log-scale was used for the Y-axis).

The diagnostic accuracy achieved by miRNAs and CA 19-9 levels, in discriminating subjects with early- and late-onset diabetes from non-diabetic HS, was assessed by the Receiver Operating Characteristic (ROC) curve analysis, with the estimation of the Area Under the ROC curve (AUC), along with its 95% confidence interval (CI), computed with 2000 stratified bootstrap replicates. Sensitivity (SE), specificity (SP), positive predictive value (PPV) and negative predictive value (NPV) were calculated at the optimal cut-off of the ROC curve, which jointly maximizes sensitivity and specificity (i.e., at the maximum Youden index). Comparison between the diagnostic accuracies achieved by each miRNA and CA 19-9 in discriminating both early-onset vs. non-diabetic HS (AUC1) and late-onset vs. non-diabetic HS (AUC2) was assessed by computing the difference from two uncorrelated AUCs (ΔAUC = AUC1-AUC2) following DeLong method (16), assuming the asymptotic normality of the AUCs estimators and setting their covariance equals to zero. The joint diagnostic ability of CA 19-9 marker and miRNA levels was assessed using individual predicted probabilities derived from a multivariable logistic regression model which included both predictors. Improvement in diagnostic ability (i.e., CA 19-9 and miRNA vs. CA 19-9 only) was assessed by comparing the AUC estimated from individual predicted probabilities derived from the two nested models (ΔAUC), following the DeLong test (16). It is worth to remark that, for both analyses, De Long test was performed on log-transformed variables. This is mainly due to the fact that the distribution of ΔAUC can be degenerate if the predictors included in the logistic model were not normally (at least symmetrically) distributed, with a consequence that the 95% CI of the difference of two nested AUCs could not have the appropriate coverage (17, 18).

A two-sided *p* < 0.05 was considered for statistical significance. All statistical analyses were performed using SAS Release 9.4 (SAS Institute, Cary, NC, USA). Data filtering, analyses and plots were carried out using R Foundation for Statistical Computing (version 3.6).

## Results

### Identification of Candidate miRNAs Associated With Recent Diabetes

The extent of expression difference between pools was preliminarily guessed by PCA (14). This highlighted that the pool sets from subjects with early-onset diabetes (Pool_4_ and Pool_9_) were spatially separated either from those including subjects with late-onset diabetes (Pool_5_ and Pool_10_) and from those constituted by non-PanC controls (Pool_1−2−3_); the PCA also revealed a close proximity between the Pool_5_ and the three pool sets from non-PanC (Pool_1−2−3_). These spatial distributions suggested a difference in miRNAs expression levels between subjects with early-onset diabetes and both the individuals with late-onset diabetes and non-PanC controls.

According to this, candidate miRNAs with the potential to discriminate subjects with early-onset diabetes from those with late-onset diabetes were identified by comparing miRNA expression levels between Pool_4−9_ and Pool_5−10._ A total of 47 miRNAs resulted differently expressed, with 29 up- and 18 down-regulated in early-onset subjects (Table 2A). The most significant changes were measured for miR-541 and miR-325. Similarly, candidate miRNAs with the potential to distinguish subjects with early-onset diabetes from non-PanC controls were identified by comparing Pool_4−9_ and Pool_1−2−3−5_. As reported in Table 2B, 33 miRNAs were over-expressed in the former group, while 25 were down-regulated; miR-4666a-3p and miR-325 exhibited the most significant difference.

**Table 2:** MicroRNA differentially expressed in pools of plasma from subjects with early-onset diabetes compared to those with late-onset diabetes (A) and to non-pancreatic cancer controls (B).

**A**	**Fold-change (95% CI)**	***P*-value**
hsa-miR-126	1.558 (1.31–1.86)	0.001
hsa-miR-4742	1.390 (1.22–1.58)	0.001
hsa-miR-4660	1.443 (1.24–1.68)	0.001
hsa-miR-9-1	1.416 (1.21–1.65)	0.001
hsa-miR-4439	1.579 (1.29–1.94)	0.001
hsa-miR-4524a-5p	1.151 (1.08–1.23)	0.001
hsa-miR-892c-5p	1.548 (1.27–1.89)	0.001
hsa-miR-3976	1.467 (1.22–1.76)	0.002
hsa-miR-1271	1.360 (1.17–1.58)	0.002
hsa-miR-302a-3p	1.433 (1.20–1.72)	0.002
hsa-miR-4263	1.797 (1.33–2.43)	0.002
hsa-miR-6807-3p	1.384 (1.15–1.66)	0.004
hsa-miR-301a	1.159 (1.07–1.26)	0.004
hsa-miR-20b	1.649 (1.24–2.20)	0.005
hsa-miR-98	1.731 (1.25–2.39)	0.005
hsa-miR-3119-2	1.312 (1.12–1.54)	0.005
hsa-miR-95-3p	1.310 (1.11–1.55)	0.006
hsa-miR-603	1.274 (1.09–1.49)	0.008
hsa-miR-29a	1.493 (1.15–1.93)	0.008
hsa-miR-6830	1.249 (1.08–1.44)	0.008
hsa-miR-490-3p	1.387 (1.12–1.71)	0.008
hsa-miR-3670-1	1.337 (1.11–1.61)	0.008
hsa-miR-3670-2	1.337 (1.11–1.61)	0.008
hsa-miR-2278	1.479 (1.15–1.91)	0.009
hsa-miR-34b-5p	1.341 (1.11–1.63)	0.009
hsa-miR-378h	1.337 (1.10–1.62)	0.009
hsa-miR-4735	1.362 (1.11–1.67)	0.009
hsa-miR-3672	1.323 (1.10–1.59)	0.009
hsa-miR-8084	1.261 (1.08–1.14)	0.010
hsa-miR-541	−1.170 (−1.23; −1.11)	<0.001
hsa-miR-325	−1.500 (−1.71; 1.31)	<0.001
hsa-miR-6733	−1.242 (−1.37; −1.12)	0.001
hsa-miR-3160-3p	−1.181 (−1.28; −1.09)	0.001
hsa-miR-320b-1	−1.231 (−1.36; −1.11)	0.002
hsa-miR-4779	−1.220 (−1.34; −1.11)	0.002
hsa-miR-8052	−1.716 (−2.30; −1.28)	0.003
hsa-miR-4503	−1.507 (−1.89; −1.20)	0.004
hsa-miR-5572	−1.510 (−1.94; −1.17)	0.006
hsa-miR-4501	−1.382 (−1.68; −1.13)	0.006
hsa-miR-548q	−1.223 (−1.38; −1.08)	0.006
hsa-miR-345	−1.453 (−1.83; −1.15)	0.006
hsa-miR-3199	−1.263 (−1.46; −1.09)	0.007
hsa-miR-1247-5p	−1.712 (−2.42; −1.21)	0.008
hsa-miR-1226-3p	−1.429 (−1.80; −1.14)	0.008
hsa-miR-221	−1.376 (−1.70; 1.11)	0.009
hsa-miR-520d-3p	−1.481 (−1.92; −1.14)	0.009
hsa-miR-6835	−1.145 (−1.25; −1.05)	0.010
hsa-miR-4666a-3p	1.334 (1.21–1.47)	<0.001
hsa-miR-4487	1.587 (1.29–1.96)	0.001
hsa-miR-6840	1.337 (1.16–1.54)	0.002
hsa-miR-490-3p	1.443 (1.20–1.73)	0.002
hsa-let-7b-5p	1.631 (1.27–2.09)	0.002
hsa-miR-4799-5p	1.239 (1.11–1.38)	0.003
hsa-miR-6084	1.287 (1.12–1.47)	0.003
hsa-miR-4694-5p	1.384 (1.16–1.65)	0.003
hsa-miR-3976	1.385 (1.16–1.66)	0.004
hsa-miR-3117-5p	1.565 (1.21–2.02)	0.004
hsa-miR-487a	1.364 (1.14–1.63)	0.004
hsa-miR-2392	1.276 (1.11–1.47)	0.004
hsa-miR-20b	1.561 (1.20–2.02)	0.005
hsa-miR-518c-3p	1.381 (1.14–1.67)	0.005
hsa-miR-6795-5p	1.563 (1.19–2.04)	0.006
hsa-miR-515-5p	1.266 (1.10–1.46)	0.006
hsa-miR-641	1.368 (1.13–1.65)	0.006
hsa-miR-4503	1.318 (1.11–1.56)	0.006
hsa-miR-18a-5p	1.969 (1.26–3.00)	0.007
hsa-miR-29a	1.423 (1.14–1.77)	0.007
hsa-miR-497-3p	1.255 (1.09–1.45)	0.007
hsa-miR-2278	1.428 (1.14–1.79)	0.007
hsa-miR-4278	1.203 (1.07–1.35)	0.007
hsa-miR-6866	1.414 (1.14–1.76)	0.007
hsa-miR-587	1.306 (1.10–1.55)	0.008
hsa-miR-3675-5p	1.283 (1.09–1.50)	0.008
hsa-miR-1279	1.541 (1.17–2.04)	0.008
hsa-miR-6807-3p	1.315 (1.10–1.57)	0.008
hsa-miR-4439	1.414 (1.13–1.77)	0.008
hsa-miR-6508-5p	1.325 (1.10–1.60)	0.009
hsa-miR-3652	1.315 (1.10–1.58)	0.009
hsa-miR-3928	1.203 (1.06–1.36)	0.009
hsa-miR-4790-5p	1.425 (1.12–1.81)	0.010
hsa-miR-325	−1.407 (−1.58; −1.25)	<0.001
hsa-miR-541	−1.131 (−1.19; −1.07)	0.001
hsa-miR-451b	−1.197 (−1.32; −1.09)	0.003
hsa-miR-4709	−1.281 (−1.45; −1.12)	0.003
hsa-miR-4290	−1.48 (−1.83; −1.20)	0.003
hsa-miR-4766	−1.153 (−1.25; −1.07)	0.003
hsa-miR-3160-3p	−1.145 (−1.23; −1.06)	0.003
hsa-miR-3911	−1.273 (−1.45; −1.12)	0.003
hsa-miR-4258	−1.972 (−2.89; −1.34)	0.004
hsa-miR-4302	−1.431 (−1.76; −1.17)	0.004
hsa-miR-7703	−1.216 (−1.36; −1.09)	0.004
hsa-miR-498	−1.310 (−1.53; −1.12)	0.005
hsa-miR-548ag	−1.428 (−1.76; −1.16)	0.005
hsa-miR-5590-3p	−1.345 (−1.61; −1.12)	0.006
hsa-miR-936	−1.305 (−1.54; −1.11)	0.006
hsa-miR-6873-3p	−1.155 (−1.26; −1.06)	0.006
hsa-miR-4779	−1.23 (−1.40; −1.08)	0.007
hsa-miR-3179	−1.297 (−1.53; −1.10)	0.007
hsa-miR-585	−1.36 (−1.66; −1.12)	0.008
hsa-miR-548y	−1.334 (−1.61; −1.11)	0.008
hsa-miR-3928-5p	−1.314 (−1.57; −1.10)	0.009
hsa-miR-1287-3p	−1.176 (−1.31; −1.06)	0.009
hsa-miR-5701	−1.247 (−1.45; −1.08)	0.010
hsa-miR-548s	−1.191 (−1.34; −1.06)	0.010
hsa-miR-1292-3p	−1.367 (−1.69; −1.11)	0.010

**Figure 1:**
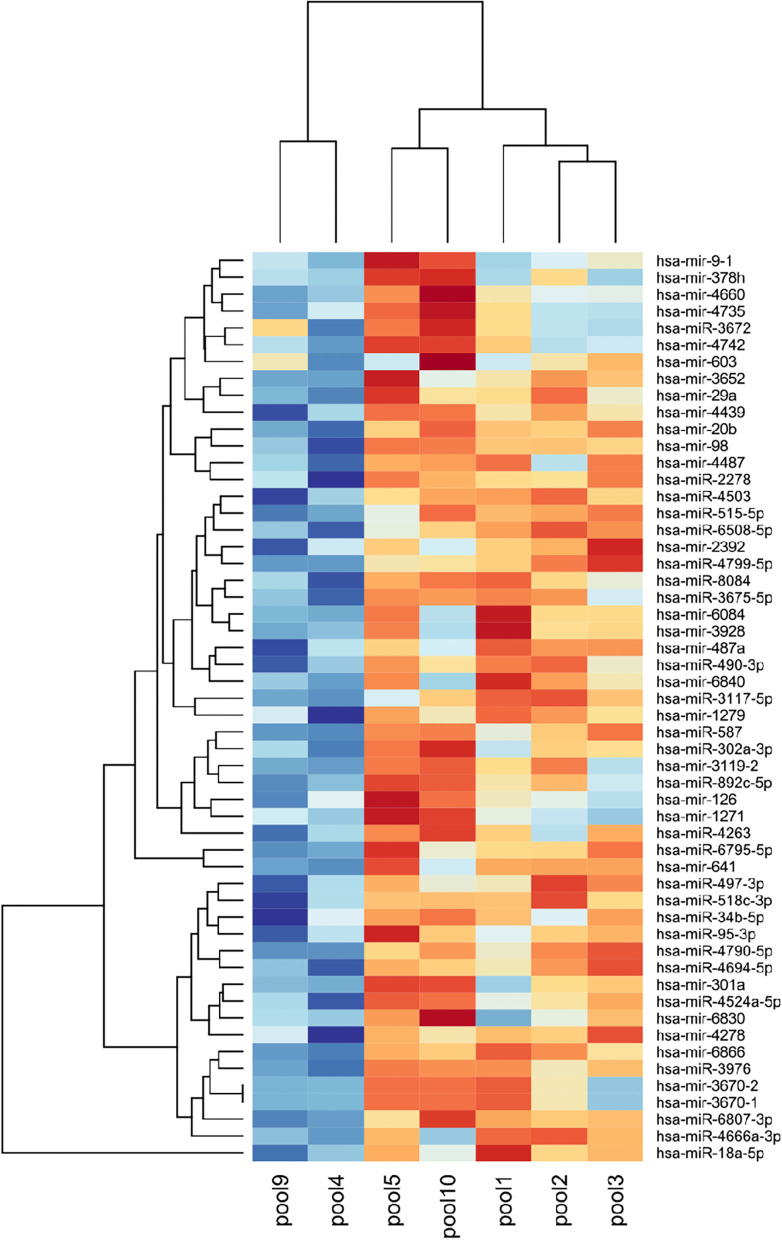
Heatmap of miRNAs identified as up-regulated by microarray in pool sets of plasma from subjects with early-onset diabetes compared to those from late-onset diabetes and controls. Blu indicates up-regulation and red indicates the down-regulation of miRNA expression. Plasma pool samples and miRNAs are listed across the x- and y-axis, respectively. Pool_1−2−3:_ sets of plasma from non-PanC controls; Pool_4_ and Pool_9_: sets of plasma from subjects with early-diabetes, non-PanC controls and PanC patients, respectively; Pool_5_ and Pool_10_: sets of plasma from subjects with late-diabetes, non-PanC controls and PanC patients, respectively.

The heatmap in Figure 1 was drawn using miRNAs whose expression values were up-regulated in early-onset diabetes subjects when compared with late-onset diabetes and non-PanC controls. The resulting clustering of pools was consistent with that observed in the preliminary PCA plot (14).

### Selection of Candidate miRNAs Associated With Recent Diabetes for Further Validation Tests

MiRNAs with a potential role in the pathogenesis of recent diabetes associated with PanC were picked among those significantly over-expressed reported in Tables 2A, B. Both these miRNA sets were prioritized by functional pertinence by means of the IPA BioProfiler tool, as previously reported (14). Following this approach, the lists of miRNAs with annotated effect on human diseases and biological functions and with reported causal or correlation evidences exisisting between miRNAs and PanC/diabetes in literature data were reduced in number to 11 and 8, respectively (Supplementary Table 1). Candidate miRNAs were further filtered according to: (i) their expression trends, namely only miRNAs that were previously reported as up-regulated were considered; (ii) the yielded effect of miRNA on PanC and diabetes, where only miRNAs reported as involved in both the diseases were considered; (iii) the fold-change values, where miRNAs showing the highest fold-change values were selected. Finally, miR-20b-5p, miR-29a, and miR-18a-5p were retained for the validation tests. In detail, miR-29a and miR-20b-5p distinguished subjects with early-onset diabetes from both those with late-onset diabetes or HS, whereas miR-18a-5p significantly discriminated only subjects with early-onset diabetes from HS.

### Validation of Candidate miRNAs Associated With Recent Diabetes

Median expression levels of miRNAs and CA 19-9 determinations in the validation cohort are shown in Table 3. To assess the association between candidate miRNAs and recent diabetes as early manifestation of PanC, differences in miRNA expression levels between subjects enrolled into the study were first evaluated by considering the entire cohorts of subjects with early- or late-diabetes (PanC + non-PanC), and then by splitting PanC patients and non-PanC controls into two independent subgroups including early- and late-diabetics, respectively.

**Table 3:** Expression levels of miRNAs and CA 19-9 in the validation cohort of subjects with early- and late-onset diabetes, and in the control group including non-diabetic healthy subjects.

	**Early diabetes**	**Late diabetes**	**Controls**	***P*-value**	***P*-value**	***P*-value**
	**(ED)**	**(LD)**		**ED vs. LD**	**ED vs. controls**	**LD vs. controls**
	**PanC** **+** **Non-PanC**	**PanC** **+** **Non-PanC**	**Non-diabetic HS**			
	**(*****N*** **=** **30)**	**(*****N*** **=** **39)**	**(*****N*** **=** **100)**			
**miR-20b-5p**	23.6 (12.4–34.0)	12.4 (1.2–39.6)	0.15 (0.15–1.0)	0.146	* <0.001*	* <0.001*
**miR-29a**	95.6 (63.6–126.4)	53.6 (9.2–154.4)	3.6 (0.9-15.2)	0.097	* <0.001*	* <0.001*
**miR-18a-5p**	29.0 (14.8–49.6)	–	1.0 (0.3–7.2)	–	* <0.001*	–
**CA 19-9**	141.0 (7.9–1138.0)	44.8 (4.8–278.0)	4.2 (2.5–8.8)	0.208	* <0.001*	* <0.001*
	**PanC (*****N*** **=** **17)**	**PanC (*****N*** **=** **23)**				
**miR-20b-5p**	28.0 (14.0–32.0)	8.6 (0.4–25.2)		*0.019*	* <0.001*	* <0.001*
**miR-29a**	100.4 (58.8–149.2)	33.4 (0.7–86.8)		*0.011*	* <0.001*	0.060
**miR-18a-5p**	27.6 (20.4–44.0)	–		–	* <0.001*	–
**CA 19-9**	887.0 (240.0–3311.0)	241.0 (64.9–397.4)		0.052	* <0.001*	* <0.001*
	**Non-PanC (*****N*** **=** **13)**	**Non-PanC (*****N*** **=** **16)**				
**miR-20b-5p**	20.8 (11.6–34.0)	22.8 (5.6–43.6)		0.719	* <0.001*	* <0.001*
**miR-29a**	89.2 (66.4–120.8)	118.0 (25.2–192.0)		0.688	* <0.001*	* <0.001*
**miR-18a-5p**	30.4 (14.8–51.2)	–		–	* <0.001*	–
**CA 19-9**	8.9 (5.3–14.3)	4.5 (3.2–8.3)		0.097	*0.030*	0.688

*Values were reported as median along with interquartile range (IQR, i.e., first-third quartiles)*.

*PanC, pancreatic cancer; Non-PanC, not-pancreatic cancer controls; HS, healthy subjects*.

*Statistically significant results (p < 0.05) are in italic*.

**Figure 2:**
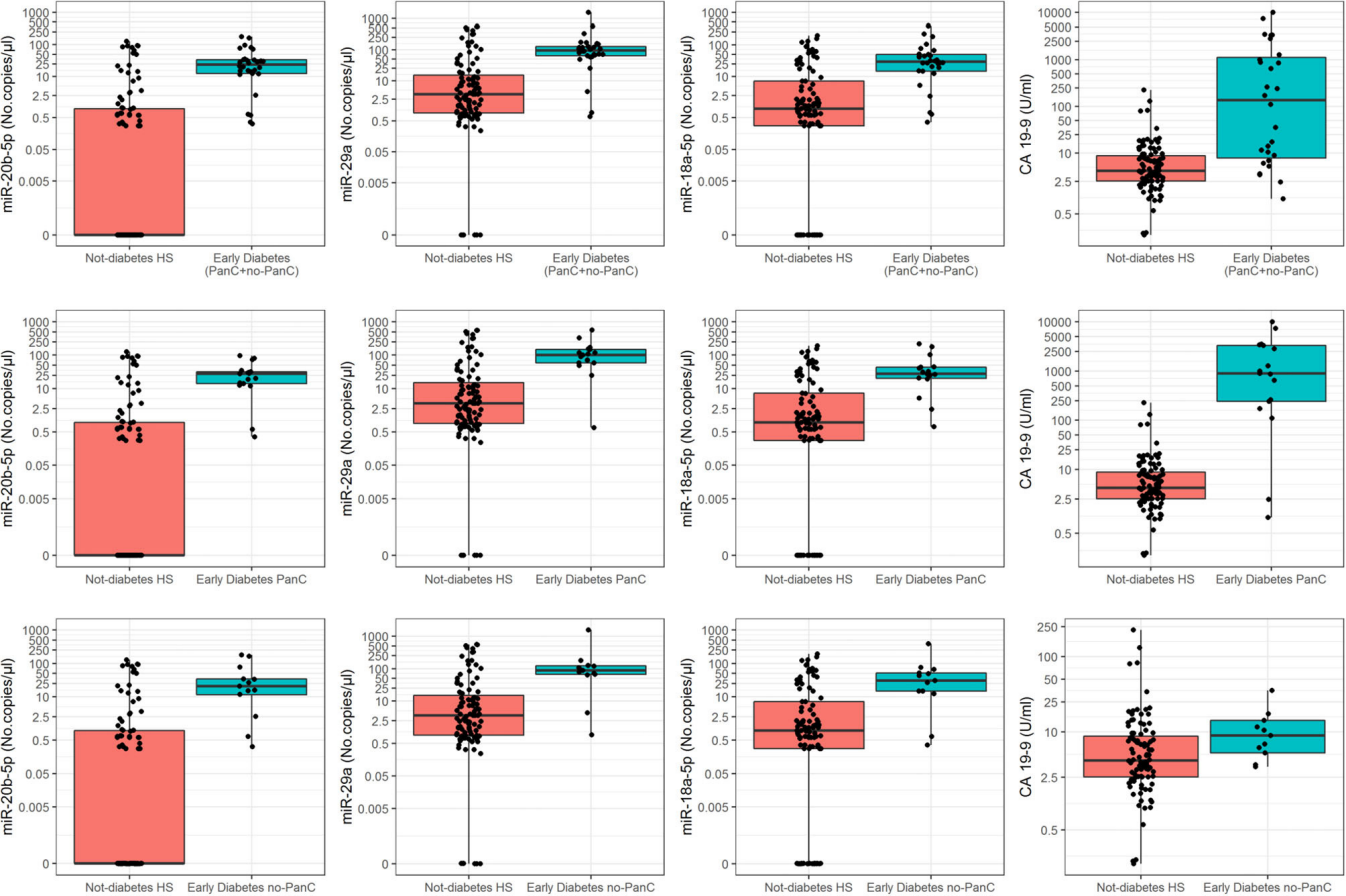
Boxplots of the absolute quantification of circulating miRNAs and CA 19-9 levels in subjects with early-onset diabetes (PanC patients + non-PanC controls) and in non-diabetic healthy subjects (HS). Values of both miRNAs (no.copies/μl) and CA 19-9 (U/ml) are reported in log scale (y-axis).

Plasma levels of miR-20b-5p and miR-29a did not significantly differ between the entire cohorts of subjects with early- (*n* = 30) and late-diabetes (*n* = 39). However, when PanC patients and non-PanC controls were split into two independent subgroups, the expression levels of miR-20b-5p and miR-29a were significantly increased in plasma from PanC patients with early- (*n* = 17) compared to those with late-diabetes (*n* = 23); conversely, no significant differences emerged between non-PanC with recent (*n* = 13) and long term diabetes (*n* = 16). On the other hand, circulating levels of miR-20b-5p, miR-29a, and miR-18a-5p were significantly increased either in the entire cohort and in the two independent subgroups of subjects with early diabetes compared to non-diabetic HS, in line with the trend of the CA 19-9 determinations (Figure 2).

As reported in Table 3, miR-20b-5p and miR-29a showed increased expression levels also in plasma from subjects with late-diabetes compared to non-diabetic HS. However, the alterations in circulating miRNAs levels were in line with the trend of the CA 19-9 determinations only in the overall population of late diabetics and in the subgroup of PanC patients with late-diabetes compared to the control group.

The median levels of miRNAs did not statistically differ between hemolyzed and not-hemolyzed samples (Supplementary Table 2), classified either according to the optical density of free hemoglobin at 414 nm and to the lipemia-independent hemolysis score (15). Thus, all the samples were included in the following evaluations.

### Diagnostic Performance of Recent Diabetes-Associated miRNAs

We evaluated the diagnostic abilities of miR-20b-5p, miR-18a-5p, and miR-29a to discriminate subjects with early-onset diabetes from the non-diabetic HS. The diagnostic power of miRNA in discriminating recent diabetes from non-diabetic HS was tested first by considering the entire cohorts of subjects with early-diabetes (PanC + non-PanC), and then by splitting PanC patients and non-PanC controls into two independent subgroups.

**Figure 3:**
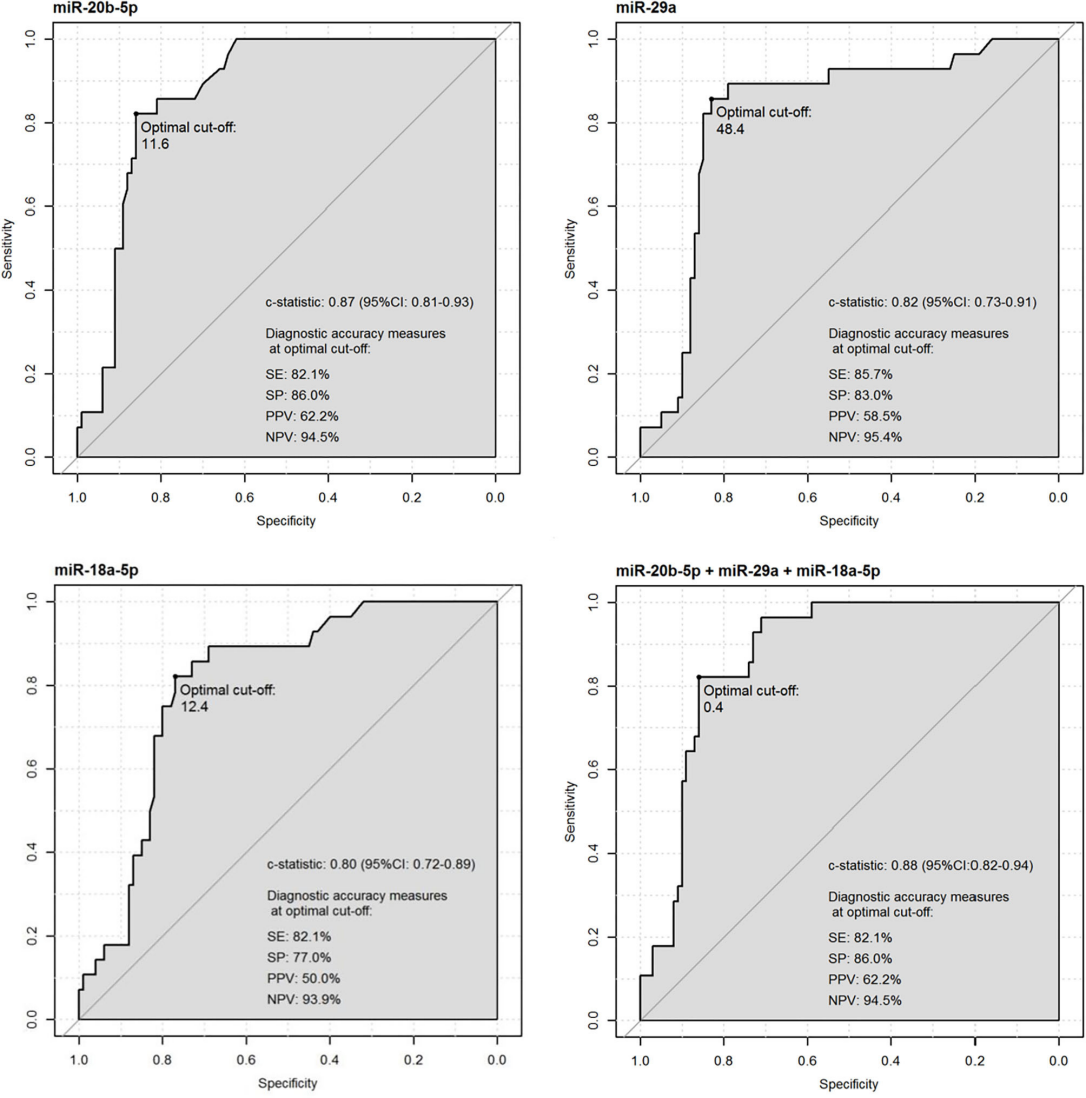
Receiver operating characteristics (ROC) curve analysis of miR-20b-5p, miR-29a, and miR-18a-5ap in discriminating subjects with early-onset diabetes (PanC patients + non-PanC controls) from non-diabetic healthy subjects.

As shown in Figure 3, every single miRNA reached a similar diagnostic performance in distinguishing subjects with recent diabetes (both PanC and non-PanC) from the non-diabetic HS. We also determined the diagnostic performance of the combination between all the three miRNAs (AUC = 0.875, 95% CI = 0.815–0.935). Noteworthy, when PanC patients and non-PanC controls were split into two independent subgroups, the diagnostic performance achieved by each miRNA in distinguishing PanC with early-onset diabetes from non-diabetic HS (miR-20b-5p: AUC = 0.877, 95% CI = 0.811–0.943; miR-29a: AUC = 0.838, 95% CI = 0.738–0.938; miR-18a-5p: AUC = 0.824, 95% CI = 0.741–0.907; miRNAs panel: AUC = 0.877, 95% CI = 0.810–0.944) was similar to the ones achieved in discriminating non-PanC with recent diabetes from non-diabetic HS (miR-20b-5p: AUC = 0.873, 95% CI = 0.798-0.947; miR-29a: AUC = 0.810, 95% CI = 0.691–0.929; miR-18a-5p: AUC = 0.785, 95% CI = 0.666-0.905 miRNAs panel: AUC = 0.866, 95% CI = 0.788–0.945).

We sought to investigate whether the expression levels of miR-20b-5p and miR-29a were also able to distinguish between subjects with late-onset diabetes and HS (data are shown in Supplementary Table 3). Althought each miRNA showed the ability to discriminate either PanC or non-PanC with late-diabetes from the HS, the measures of diagnostic accuracy reached in distinguishing PanC with late-diabetes from HS were significantly lower than those achieved in discriminating between PanC with early-diabetes and the HS (miR-20b-5p: AUC = 0.760, 95% CI = 0.655-0.865 vs. AUC = 0.877, 95% CI = 0.811–0.943; miR-29a: AUC = 0.630, 95% CI = 0.479–0.781 vs. AUC = 0.838, 95% CI = 0.738–0.938). Conversely, no significant differences emerged between the discriminatory ability for either non-PanC with early or late-diabetes from HS.

### Diagnostic Performance of Recent Diabetes-Associated miRNAs Compared to the CA 19-9

We next evaluate the diagnostic abilities of miR-20b-5p, miR-18a-5p, and miR-29a to the one achieved by CA 19-9 serum marker to discriminate subjects with early-onset diabetes from non-diabetic HS, and improvement of the diagnostic performance achieved by the two biomarkers (i.e., CA 19-9 + miRNA) with respect to CA 19-9 alone was also assessed. To pursue these intents, PanC patients and non-PanC controls were split into two independent subgroups of subjects with early-onset diabetes.

Data are shown in Table 4. The diagnostic performance of serum CA 19-9 levels in discriminating PanC with recent diabetes from non-diabetic HS (AUC = 0.898, 95% CI = 0.763–1.000) was similar to the ones achieved by each single miRNA (miR-20b-5p: AUC = 0.877, 95% CI = 0.811–0.943; miR-29a: AUC = 0.838, 95% CI = 0.738–0.938; miR-18a-5p: AUC = 0.824, 95% CI = 0.741–0.907). In addition, the operating characteristics achieved by combining each miRNA to the CA 19-9 determinations (miRNA and CA 19-9 jointly considered) were not significantly higher than the one achieved by the serum CA 19-9 marker tested alone.

**Table 4:** The diagnostic performance of miRNAs and CA 19-9 levels, tested independently and in combination using a logistic regression model, in discriminating either pancreatic cancer (PanC) or non-PanC controls with recent-diabetes from non-diabetic healthy subjects.

**Early- no PanC vs. Non-diabetic HS**		**CA 19-9**	**miRNA**	**CA 19-9 + miRNA**	***p*-value**
**miR-20b-5p**	C-statistic (95% CI)^*^	0.700 (0.573–0.827)	0.868 (0.786–0.950)	0.900 (0.842–0.958)	**0.003**
	Optimal cutoff value^§^	35.7	2.6	0.132^∧^	
	SE (%)	100%	81.8%	100%	
	SP (%)	40.0%	81.0%	81.0%	
	Overall accuracy (%)	45.9%	81.1%	82.9%	
	PPV (%)	15.5%	32.1%	36.7%	
	NPV (%)	100%	97.6%	100%	
**miR-29a**	C-statistic (95% CI)^*^		0.797 (0.662–0.933)	0.846 (0.752–0.941)	0.076
	Optimal cutoff value^§^		63.6	0.142^∧^	
	SE (%)		81.8%	81.8%	
	SP (%)		85.0%	81.0%	
	Overall accuracy (%)		84.7%	81.1%	
	PPV (%)		37.5%	32.1%	
	NPV (%)		97.7%	97.6%	
**miR-18a-5p**	C-statistic (95% CI)^*^		0.774 (0.639–0.909)	0.847 (0.760–0.934)	0.055
	Optimal cutoff value^§^		12.4	0.152^∧^	
	SE (%)		81.8%	81.8%	
	SP (%)		77.0%	83.0%	
	Overall accuracy (%)		77.5%	82.9%	
	PPV (%)		28.1%	34.6%	
	NPV (%)		97.5%	97.6%	
**Early-PanC vs. Non-diabetic HS**					
**miR-20b-5p**	C-statistic (95% CI)^*^	0.898 (0.763–1.000)	0.877 (0.811–0.943)	0.972 (0.933–1.000)	0.143
	Optimal cutoff value^§^	110.6	12.0	0.356^∧^	
	SE (%)	88.2%	88.2%	88.2%	
	SP (%)	98.0%	86.0%	98.0%	
	Overall accuracy (%)	96.6%	86.3%	96.6%	
	PPV (%)	88.2%	51.7%	88.2%	
	NPV (%)	98.0%	97.7%	98.0%	
**miR-29a**	C-statistic (95% CI)^*^		0.838 (0.738–0.938)	0.956 (0.894–1.000)	0.140
	Optimal cutoff value^§^		24.8	0.447^∧^	
	SE (%)		94.1%	88.2%	
	SP (%)		79.0%	99.0%	
	Overall accuracy (%)		81.2%	97.4%	
	PPV (%)		43.2%	93.8%	
	NPV (%)		98.8%	98.0%	
**miR-18a-5p**	C-statistic (95% CI)^*^		0.824 (0.741–0.907)	0.949 (0.880–1.000)	0.145
	Optimal cutoff value^§^		2.4	0.365^∧^	
	SE (%)		94.1%	88.2%	
	SP (%)		69.0%	98.0%	
	Overall accuracy (%)		72.6%	96.6%	
	PPV (%)		34.0%	88.2%	
	NPV (%)		98.6%	98.0%	

*CI, confidence interval; SE, sensibility; SP, specificity. PPV, positive predictive value; NPV: negative predictive value. SE. SP. PPV and NPV measures were calculated at the optimal cut-offs*.

* *c-statistics were calculated on the basis of estimated predicted probabilities from logistic models*.

§ *optimal decision threshold for CA 19-9, miRNA expressions and predicted probabilities which jointly*.

*maximize sensibility and specificity (i.e., max Youden index)*.

∧ *optimal decision threshold for predicted probabilities estimated from multivariable logistic models*.

# *p-values from DeLong test to compare c-statistics for nested models (i.e., CA 19-9 + miRNA vs. CA 19-9 models). Statistically significant results (p < 0.05) are in bold*.

**Figure 4:**
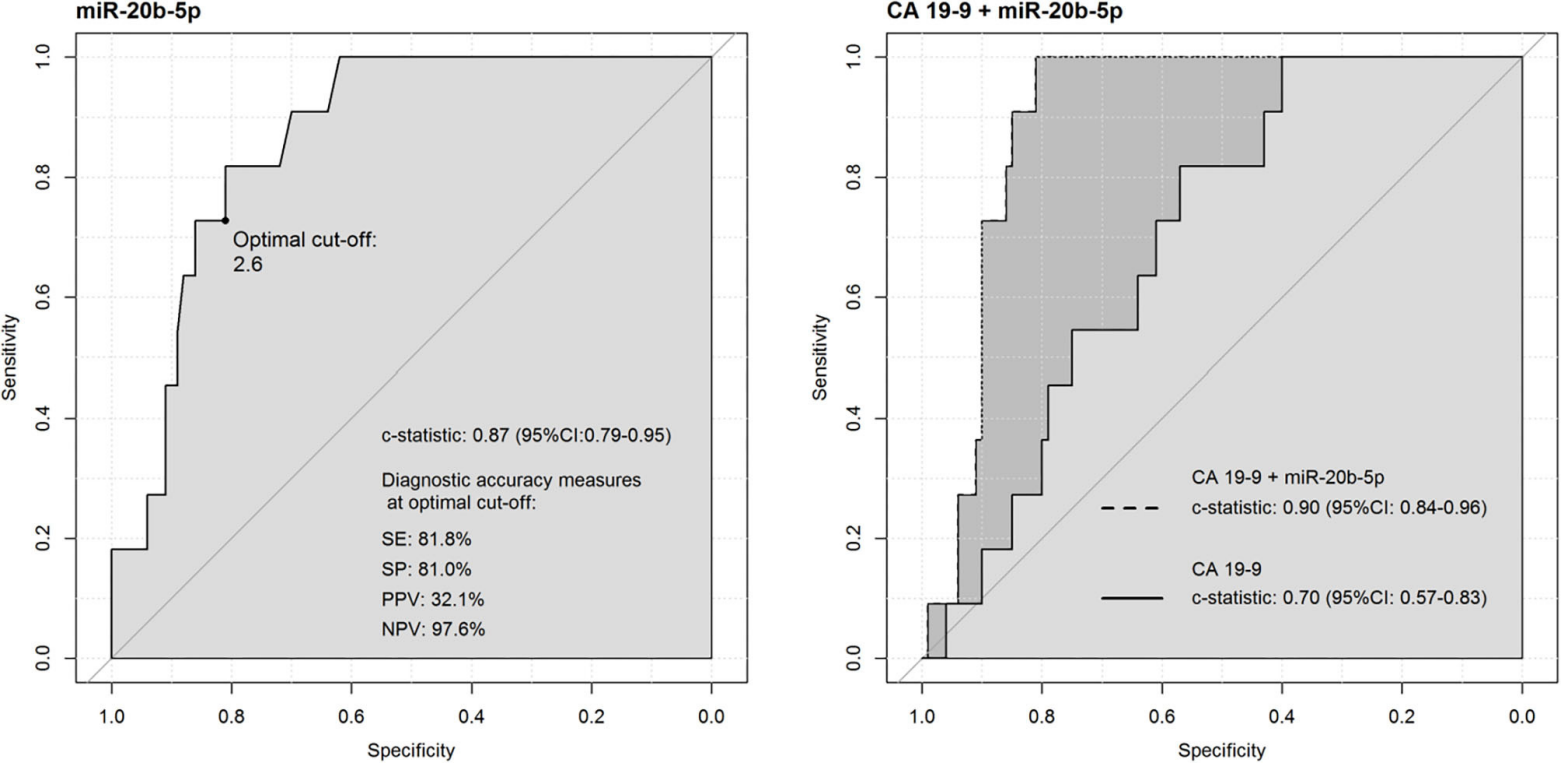
Receiver operating characteristics (ROC) curve analysis of miR-20b-5p in discriminating non-PanC with recent diabetes from non-diabetic healthy subjects, compared to the CA 19-9 determinations.

As for the diagnostic abilities of miRNAs to discriminate between non-PanC with recent diabetes from the HS without diabetes, a less than acceptable discriminatory power was achieved by serum CA 19-9 levels (AUC = 0.700, 95% CI = 0.573–0.827), and by both miR-18a-5p (AUC = 0.774, 95% CI = 0.639–0.909) and miR-29a (AUC = 0.797, 95% CI = 0.662–0.933). Conversely, the diagnostic performance achieved by the miR-20b-5p alone was fairly good (AUC = 0.868, 95% CI = 0.786–0.950), and higher than the one achieved by the serum CA 19-9 marker tested alone. The SP and PPV values determined by the ROC curve of miR-20b-5p were higher than those derived from the ROC curve of the CA 19-9 markers (SP = 81%, PPV = 32.1%, and SP = 40.0%, PPV = 15.5%, respectively). The operating characteristics were further improved by combining the levels of miR-20b-5p with the CA 19-9 determinations: the joint discrimination ability of the two markers (AUC = 0.900, 95% CI = 0.842–0.958) was significantly higher than the one achieved by the serum CA 19-9 marker tested alone (*p* = 0.003), and the highest discriminatory power as determined by the ROC curve was reached with the cut-off value of 0.132 (i.e., relative to predicted probabilities) used to classify the non-diabetic HS (subjects below the cut-off) and the non-PanC with recent diabetes (subjects above the cut-off) Figure 4.

## Discussion

Despite the clinical utility of CA19-9 for predicting PanC survival after surgery and clinical treatment, the use of this tumor marker for diagnostic decision reflects a low positive predictive value (19–21). Identification of new potential targets for early PanC could reduce the morbidity rate, and dissecting recent-onset diabetes as a manifestation of PanC is likely to give important insights into this issue. Herein, we aimed to uncover for circulating miRNAs a role in recent diabetes as potential early manifestation of PanC. The hypothesis is that the recognition of recent PanC-associated diabetes could enrich the population at high-risk for PanC development, up to define a marker for the detection of precocious symptomatic cancer.

Candidate miRNAs associated with recent diabetes were picked among miRNAs significantly over-expressed in pools from subjects with recent diabetes compared to either those with late-onset diabetes and no-PanC controls. After bioinformatic evaluation miR-29a, miR-20b-5p (increased in pools from subjects with recent diabetes compared to either those with late-onset diabetes or no-PanC) and miR-18a-5p (increased in pools from subjects with early-onset diabetes compared to no-PanC) were retained for the validation tests. The ddPCR did not confirm the differential expression of miR-20b-5p and miR-29a in plasma from subjects with early- and late diabetes within the validation cohort. However, when PanC patients and no-PanC controls with recent diabetes were split into two independent subgroups, the levels of miR-20b-5p and miR-29a were significantly increased in plasma from PanC patients with recent diabetes compared to those with late-onset diabetes, suggesting the potential association between the circulating levels of miR-20b-5p and miR-29a and the diabetes of recent diagnosis in PanC. In addition, plasma levels of miR-20b-5p, miR-29a, and miR-18a-5p were significantly over-expressed in the validation cohort of subjects with recent diabetes compared to non-diabetic HS, and persisted in PanC patients and non-PanC controls with early-onset diabetes split into independent subgroups. Accordingly, the diagnostic performance achieved by miR-20b-5p, miR-29a, and miR-18a-5p in distinguishing PanC with early-onset diabetes from non-diabetic HS was similar to the ones achieved by each miRNA in discriminating non-PanC with recent-diabetes from the HS without diabetes. It is worth to note that the increase of miR-20b-5p and miR-29 levels persisted in PanC patients and non-PanC controls with late-diabetes compared to the HS in the validation cohort. However, each miRNA achieved a higher diagnostic accuracy in distinguishing early-diabetes rather than late-diabetes from the HS poluation within the PanC patients. These findings suggested the association between miR-18a-5p and early diabetes, and highlighted that circulating levels of miR-20b-5p and miR-29 could be useful for identifying early diabetes in PanC, albeit not as an early manifestation of cancer.

Alterations in circulating levels of miR-18a-5p, miR-20b-5p, and miR-29a in subjects with recent diabetes compared to non-diabetic HS were in line with CA 19-9 determinations, thus the diagnostic abilities of miRNAs were also evaluated in comparison to the CA 19-9 tumor marker. Serum CA19-9 levels have been reported to be increased and correlated with glycemic status in otherwise normal diabetic subjects, whereas the levels of CA 19-9 in patients with PanC should be interpreted with regard to both the malignant and the diabetes status (22, 23). Therefore, to pursue our intent, the cohort of subjects with early-onset diabetes was split into two independent subgroups including PanC or non-PanC, respectively. As described in the results section, the diagnostic performance of serum CA 19-9 levels in discriminating PanC patients with recent-diabetes from non-diabetic HS was moderately good (AUC = 0.898), and similar to the diagnostic power reached by every single miRNA. Conversely, the CA 19-9 determinations achieved a less than acceptable discriminatory power in differentiating non-PanC with recent-diabetes from the HS without diabetes (AUC = 0.700), and the best diagnostic performance was achieved by miR-20b-5p (AUC = 0.868), with a gain in the specificity value and the positive predictive value resulting in a lower false-positive rate in respect to the determination of the conventional marker (SP = 81%; PPV = 32.1% and SP = 40.0%; PPV = 15.5%, respectively). In addition, miR-20b-5p plasma levels in combination with the CA 19-9 determinations further improved the operating characteristics compared to the CA 19-9 marker tested alone (AUC = 0.900; SP = 81%; PPV = 36.7%; *p* = 0.003). These findings highlighted for miR-20b-5p an exclusive role as a more informative marker in distinguishing non-PanC with recent diabetes from the non-diabetic HS compared to the CA 19-9.

The literature provides evidences of the disruption of miR-20b-5p, miR-18a-5p, and miR-29a circulating levels in diabetes (24–29), and a role for these miRNAs in the pathogenesis of the disease and in the diabetic complications has been documented (27, 28, 30–33). Conflicting data emerged about the alteration of miR-20b-5p in blood from diabetic subjects (24–26), and its over-expression in primary human skeletal muscle cells was reported to influence both the basal glycogen synthesis and the insulin-stimulated glycogen accumulation (25). The elevation of miR-18a-5p levels in blood was found to correlate with the risk of type 2 diabetes and impaired fasting glucose, and a role for this miRNA as an independent positive predictor for insulin resistance has been uncovered (29). Decreased levels of miR-18a-5p were found in plasma from pre-diabetic individuals without subsequent diabetes occurrence compared to either those with normal glucose tolerance or diabetic patients (34). Evidences also exist about the increase of miR-29a circulating levels in diabetics and pre-diabetics subjects compared to controls (27, 28), and a positive association between miR-29a and the levels of the glycated hemoglobin A1c has been previously descrived (35). This miRNA is involved in the pathogenesis of diabetes mellitus by influencing the beta-cells proliferation and the insulin secretion, and a role for miR-29a over-expression in insulin resistance has been also described (36–38). Over-expression of miR-29a was also reported to cause a decrease in levels of the GLUT4 glucose transporter and to play a role in the regulation of the insulin-stimulated glucose metabolism and lipid oxidation (39–42).

Our findings on miR-18a-5p and miR-29a were in agreement with those described in previous studies where miRNA circulating levels in diabetes were tested by qRT-PCR. Noteworthy, miR-29a has been previously included in a panel of six serum miRNAs enabling the discrimination of PanC patients with diabetes from either healthy controls and non-cancer diabetics (13). Despite the authors did not associate to the miR-29a tested alone the potential association between miRNA and recent diabetes in PanC, our data confirm for this miRNA a role as biomarker for PanC-associated diabetes. As for miR-20b, our data were in line with those obtained by other authors in the exosomes isolated from patients with type 2 diabetes mellitus (25, 26), while the discordance with the direction of miRNA alteration reported by Zampetaki and colleagues might be related to differences in sample size, in methods used to retro-transcribe the RNA before the amplification step, or in the overall technologies used for miRNA quantification (24). Noteworthy, there are evidence that miR-29a expression levels are increased in skeletal muscle tissue samples from patients with type 2 diabetes (40). However, no data is available about miR-20b and miR-18a-5p expression levels in diabetes related tissue thus we were not able to assert for these miRNAs whether the circulating levels reflected the levels in the tissues.

Both miR-20b-5p and miR-29b have been previously linked also to the PanC. Briefly, high levels of miR-20b-5p in PanC tissues have been observed in patients treated with the angiogenesis inhibitor emodin (43). Similarly, the miR-29 family was widely expressed in PC (44), and played a role in the response of PanC cells to treatment by inducing resistance to gemcitabine via activation of the Wnt/β-catenin pathway (45). Several studies have demonstrated that the function of miR-29 in cancer both as a tumor suppressor and oncogene may be context-dependent (46). As to PanC, miR-29a plays an oncogenic role, as it is up-regulated in PanC cell lines and tissues, and promotes tumor cell proliferation and the mesenchymal-epithelial transition phenotype by modulating the expression of Tristetraprolin (46) the mRNA-destabilizing protein previously identified as a target of miR-29a (47).

The main findings of this study concern the potential of circulating miR-20b-5p and miR-29a for identifing early diabetes in PanC, albeit not as an early manifestation of cancer. We also highlighted for the first time a role for miR-20b-5p as a more informative marker in distinguishing non-PanC with recent diabetes from the HS without diabetes compared to the CA 19-9. A limitation of this study is its small size and the use of samples collected in a single center. This implies that our finding should be replicated in larger cohorts of subjects with early diabetics and healthy controls. Furthermore, a multicenter study will represent a more promising strategy to evaluate the potential clinical significance of miRNA plasma levels in diabetes of recent diagnosis. In addition, the role of miR-20b in diabetes remains to be fully elucidated. Thus, functional studies should be performed to shed light on the miRNA-mediated regulation of insulin production and secretion, up to the pathways driving proliferation, survival, and destruction of the β-cell. Another strength of our study is represented by the fact that the overall methodology described in our study can be used in future investigation aimed to identify other miRNAs altered in PanC-associated diabetes: miRNAs with a role as markers of early, asymptomatic PanC could be used to implement a screening strategy based on new-onset diabetes as first filter for PanC screening program.

In conclusion, although further investigations are needed to validate the biological mechanism of miR-20b-5p, miR-29a, and miR-18a-5p in the landscape of early-onset diabetes in patients with PanC, and to identify miRNAs specifically associated with recent diabetes caused by PanC, this study highlighted for circulating miRNAs a role as a potential clues to the identification of recent diabetes-associated miRNAs in PanC.

## Data Availability Statement

Publicly available datasets were analyzed in this study. This data can be found here: https://pubmed.ncbi.nlm.nih.gov/32117716/ ArrayExpress (accession number: E-MTAB-8378).

## Ethics Statement

The studies involving human participants were reviewed and approved by Ethics Committee of the Fondazione IRCCS Casa Sollievo della Sofferenza. The patients/participants provided their written informed consent to participate in this study.

## Author Contributions

FT conceived the study and wrote the manuscript. DG performed the experiments. TM and TB performed the bioinformatics analyses. MC and OP performed the previous microarray experiments and deposited data set. AF performed the statistical analysis. AA critically revised the manuscript. All the authors revised and approved the manuscript.

## Conflict of Interest

The authors declare that the research was conducted in the absence of any commercial or financial relationships that could be construed as a potential conflict of interest.

## Supplementary Material

Supplementary Table 1 BioProfiler analysis for selection of candidate miRNAs associated with recent-onset diabetes.

Supplementary Table 2 miRNAs levels in plasma with low-grade hemolysis compared to not hemolysed plasma samples.

Supplementary Table 3 Receiver operating characteristics (ROC) curve analysis of miR-20b-5p and miR-29a in discriminating subjects with late-onset diabetes (PanC patients or non-PanC controls) from non-diabetic healthy subjects.

## References

[1] Chari ST. Detecting pancreatic cancer early: problems and prospects. Semin Oncol. (2007) 34:284–94. 10.1053/j.seminoncol.2007.05.005 17674956 PMC2680914

[2] Pannala R Leirness JB Bamlet WR Basu A Petersen GM Chari ST. Prevalence and clinical profile of pancreatic cancer associated diabetes mellitus. Gastroenterology. (2008) 134:981–7. 10.1053/j.gastro.2008.01.039 18395079 PMC2323514

[3] Huxley R Ansary-Moghaddam A Berrington de González A Barzi F Woodward M. Type-II diabetes and pancreatic cancer: a meta-analysis of 36 studies. Br J Cancer. (2005) 92:2076–83. 10.1038/sj.bjc.6602619 15886696 PMC2361795

[4] Li D Tang H Hassan MM Holly EA Bracci PM Silverman DT. Diabetes and risk of pancreatic cancer: a pooled analysis of three large case-control studies. Cancer Causes Control. (2011) 22:189–97. 10.1007/s10552-010-9686-3 21104117 PMC5312666

[5] Ben Q Cai Q Li Z Yuan Y Ning X Deng S. The relationship between new-onset diabetes mellitus and pancreatic cancer risk: a case-control study. Eur J Cancer. (2011) 47:248–54. 10.1016/j.ejca.2010.07.010 20709528

[6] Pannala R Basu A Petersen GM Chari ST. New-onset diabetes: a potential clue to the early diagnosis of pancreatic cancer. Lancet Oncol. (2009) 10:88–95. 10.1016/S1470-2045(08)70337-1 19111249 PMC2795483

[7] Cui Y Andersen DK. Diabetes and pancreatic cancer. Endocr Relat Cancer. (2012) 19:F9–26. 10.1530/ERC-12-0105 22843556

[8] Li D. Diabetes and pancreatic cancer. Mol Carcinog. (2012) 51:64–74. 10.1002/mc.20771 22162232 PMC3238796

[9] He XY Li JF Yao WY Yuan YZ. Resolution of new-onset diabetes after radical pancreatic resection predicts long-term survival in patients with pancreatic ductal cell adenocarcinoma. Ann Surg Oncol. (2013) 20:3809–16. 10.1245/s10434-013-3095-2 23943021

[10] Kang SP Saif MW. Clinical Outcome of Pancreatic Cancer Patients With Diabetes Mellitus: Is Diabetes a Poor Prognostic Factor? Highlights from the “2010 ASCO Annual Meeting”. Chicago, IL: JOP. (2010) 11:334–5. 20601806

[11] Cannon RM LeGrand R Chagpar RB Ahmad SA Mc-Claine R Kim HJ. Multi-institutional analysis of pancreatic adenocarcinoma demonstrating the effect of diabetes status on survival after resection. HPB. (2012) 14:228–35. 10.1111/j.1477-2574.2011.00432.x 22404260 PMC3371208

[12] Villard A Marchand L Thivolet C Rome S. Diagnostic value of cell-free circulating micrornas for obesity and type 2 diabetes: a meta-analysis. J Mol Biomark Diagn. (2015) 6:251. 10.4172/2155-9929.1000251 27308097 PMC4905583

[13] Dai X Pang W Zhou Y Yao W Xia L Wang C.. Altered profile of serum microRNAs in pancreatic cancer-associated new onset diabetes mellitus. J Diabetes. (2016) 8:422–433. 10.1111/1753-0407.12313 25991015

[14] Mazza T Gioffreda D Fontana A Biagini T Carella M Palumbo O. Clinical significance of circulating miR-1273g-3p and miR-122-5p in Pancreatic Cancer. Front Oncol. (2020) 10:44. 10.3389/fonc.2020.00044 32117716 PMC7010806

[15] Tavano F Gioffreda D Valvano MR Palmieri O Tardio M Latiano TP. Droplet digital PCR quantification of miR-1290 as a circulating biomarker for pancreatic cancer. Sci Rep. (2018) 8:16389. 10.1038/s41598-018-34597-z 30401891 PMC6219528

[16] DeLong ER DeLong DM Clarke-Pearson DL Comparing the areas under two or more correlated receiver operating characteristic curves: a nonparametric approach. Biometrics. (1988) 44:837–45. 3203132

[17] Demler OV Pencina MJ D'Agostino RB Sr. Misuse of DeLong test to compare AUCs for nested models. Stat Med. (2012) 31:2577–87. 10.1002/sim.5328 22415937 PMC3684152

[18] Demler OV Pencina MJ Cook NR D'Agostino RB Sr. Asymptotic distribution of ΔAUC, NRIs, and IDI based on theory of U-statistics. Stat Med. (2017) 36:3334–60. 10.1002/sim.7333 28627112 PMC5931715

[19] Kim JE Lee KT Lee JK Paik SW Rhee JC Choi KW. Clinical usefulness of carbohydrate antigen 19-9 as a screening test for pancreatic cancer in an asymptomatic population. J Gastroenterol Hepatol. (2004) 19:182–6. 10.1111/j.1440-1746.2004.03219.x 14731128

[20] Boeck S Stieber P Holdenrieder S Wilkowski R Heinemann V. Prognostic and therapeutic significance of carbohydrate antigen 19-9 as tumor marker in patients with pancreatic cancer. Oncology. (2006) 70:255–64. 10.1159/000094888 16899980

[21] Goonetilleke KS Siriwardena AK. Systematic review of carbohydrate antigen (CA 19-9) as a biochemical marker in the diagnosis of pancreatic cancer. Eur J Surg Oncol. (2007) 33:266–70. 10.1016/j.ejso.2006.10.004 17097848

[22] Esteghamati A Hafezi-Nejad N Zandieh A Sheikhbahaei S Emamzadeh-Fard S Nakhjavani M. CA 19-9 is associated with poor glycemic control in diabetic patients: role of insulin resistance. Clin Lab. (2014) 60:441–7. 10.7754/clin.lab.2013.121243 24697121

[23] Uygur-Bayramicli O Dabak R Orbay E Dolapcioglu C Sargin M Kilicoglu G. Type 2 diabetes mellitus and CA 19-9 levels. World J Gastroenterol. (2007) 13:5357–9. 10.1097/MAJ.0b013e3181f0e2a0 17879406 PMC4171326

[24] Zampetaki A Kiechl S Drozdov I Willeit P Mayr U Prokopi M. Plasma microRNA profiling reveals loss of endothelial miR-126 and other microRNAs in type 2 diabetes. Circ Res. (2010) 107:810–7. 10.1161/CIRCRESAHA.110.226357 20651284

[25] Katayama M Wiklander OPB Fritz T Caidahl K El-Andaloussi S Zierath JR. Circulating exosomal miR-20b-5p is elevated in type 2 diabetes and could impair insulin action in human skeletal muscle. Diabetes. (2019) 68:515–26. 10.2337/db18-0470 30552111

[26] Xiong Y Chen L Yan C Zhou W Endo Y Liu J. Circulating exosomal miR-20b-5p Inhibition restores Wnt9b signaling and reverses diabetes-associated impaired wound healing. Small. (2020) 16:e1904044. 10.1002/smll.201904044 31867895

[27] Kong L Zhu J Han W Jiang X Xu M Zhao Y. Significance of serum microRNAs in pre-diabetes and newly diagnosed type 2 diabetes: a clinical study. Acta Diabetol. (2011) 48:61–9. 10.1007/s00592-010-0226-0 20857148

[28] Karolina Ds Armugam A Tavintharan S Wong MT Lim SC Sum CF. MicroRNA 144 impairs insulin signaling by inhibiting the expression of insulin receptor substrate 1 in type 2 diabetes mellitus. PLoS ONE. (2011) 6:e22839. 10.1371/journal.pone.0022839 21829658 PMC3148231

[29] Wang SS Li YQ Liang YZ Dong J He Y Zhang L. Expression of miR-18a and miR-34c in circulating monocytes associated with vulnerability to type 2 diabetes mellitus and insulin resistance. J Cell Mol Med. (2017) 21:3372–80. 10.1111/jcmm.13240 28661068 PMC5706576

[30] Wang X Lin B Nie L Li P. microRNA-20b contributes to high glucose-induced podocyte apoptosis by targeting SIRT7. Mol Med Rep. (2017) 16:5667–74. 10.3748/wjg.v13.i40.5357 28849008

[31] Liang GW Song Y Shao DH Xu X He ML The change of serum miR-375 and miR-29a and their correlation with glycemic control and lipid profile in patients with newly diagnosed type 2 diabete. Chin. J. Lab. Diagn. (2013) 17:475–8.

[32] Qin B Liu J Liu S Li B Ren J. MiR-20b targets AKT3 and modulates vascular endothelial growth factor-mediated changes in diabetic retinopathy. Acta Biochim Biophys Sin. (2016) 48:732–40. 10.1093/abbs/gmw065 27421659

[33] Chien HY Chen CY Chiu YH Lin YC Li WC. Differential microRNA profiles predict diabetic nephropathy progression in Taiwan. Int. J. Med. Sci. (2016) 13:457–65. 10.7150/ijms.15548 27279796 PMC4893561

[34] de Candia P Spinetti G Specchia C Sangalli E La Sala L Uccellatore A. A unique plasma microRNA profile defines type 2 diabetes progression. PLoS ONE. (2017) 12:e0188980. 10.1371/journal.pone.0188980 29200427 PMC5714331

[35] Akerman L Casas R Ludvigsson J Tavira B Skoglund C vanWijnen A. Serum miRNA levels are related to glucose homeostasis and islet autoantibodies in children with high risk for type 1 diabetes. PLoS ONE. (2018) 13:e0191067. 10.1371/journal.pone.0191067 29346396 PMC5773164

[36] Bagge A Clausen TR Larsen S Ladefoged M Rosenstierne MW Larsen L MicroRNA-29a is up-regulated in b-cells by glucose and decreases glucose-stimulated insulin secretion. Biochem Biophys Res Commun. (2012) 426:266–72. 10.1016/j.bbrc.2012.08.082 22940552

[37] Pullen TJ da Silva Xavier G Kelsey G Rutter GA. MiR-29a and miR-29b contribute to pancreatic -cell-specific silencing of monocarboxylate transporter 1 (Mct1). Mol Cell Biol. (2011) 31:3182–94. 10.1128/MCB.01433-10 21646425 PMC3147603

[38] Yang WM Jeong HJ Park SY Lee W. Induction of miR-29a by saturated fatty acids impairs insulin signaling and glucose uptake through translational repression of IRS-1 in myocytes. FEBS Lett. (2014) 588:2170–6. 10.1016/j.febslet.2014.05.011 24844433

[39] Zhou Y Gu P Shi W Li J Hao Q Cao X. MicroRNA-29a induces insulin resistance by targeting PPAR?? in skeletal muscle cells. Int J Mol Med. (2016) 37:931–8. 10.3892/ijmm.2016.2499 26936652 PMC4790643

[40] Massart J Sjögren RJ Lundell LS Mudry JM Franck N O'Gorman DJ. Altered miRNA-29 expression in type 2 diabetes influences glucose and lipid metabolism in skeletal muscle. Diabetes. (2017) 66:1807–18. 10.2337/db17-0141 28404597

[41] Plaisance V Waeber G Regazzi R Abderrahmani A. Role of microRNAs in Islet Beta-cell compensation and failure during diabetes. J. Diabetes Res. (2014) 2014:61865290. 10.1155/2014/618652 24734255 PMC3964735

[42] Williams MD Mitchell GM. MicroRNAs in insulin resistance and obesity. Exp. Diabetes Res. (2012) 2012:484696. 10.1155/2012/484696 22851965 PMC3407629

[43] Lin SZ Xu JB Ji X Chen H Xu HT Hu P. Emodin inhibits angiogenesis in pancreatic cancer by regulating the transforming growth factor-β/drosophila mothers against decapentaplegic pathway and angiogenesis-associated microRNAs. Mol Med Rep. (2015) 12:5865–71. 10.3892/mmr.2015.4158 26238071

[44] Słotwiński R Lech G Słotwińska SM. MicroRNAs in pancreatic cancer diagnosis and therapy. Cent Eur J Immunol. (2018) 43:314–24. 10.5114/ceji.2018.80051 30588176 PMC6305615

[45] Nagano H Tomimaru Y Eguchi H Hama N Wada H Kawamoto K. Microrna-29a induces resistance to gemcitabine through the wnt/beta-catenin signaling pathway in pancreatic cancer cells. Int J Oncol. (2013) 43:1066–72. 10.3892/ijo.2013.2037 23900458

[46] Sun XJ Liu BY Yan S Jiang TH Cheng HQ Jiang HS. MicroRNA-29a promotes pancreatic cancer growth by inhibiting tristetraprolin. Cell Physiol Biochem. (2015) 37:707–18. 10.1159/000430389 26356262

[47] Gebeshuber CA Zatloukal K Martinez J. Mir-29a suppresses tristetraprolin, which is a regulator of epithelial polarity and metastasis. EMBO Rep. (2009) 10:400–5. 10.1038/embor.2009.9 19247375 PMC2672883

